# Assessment of Antibiotic Resistance Among Isolates of *Klebsiella* spp. and *Raoultella* spp. in Wildlife and Their Environment from Portugal: A Positive Epidemiologic Outcome

**DOI:** 10.3390/pathogens14010099

**Published:** 2025-01-20

**Authors:** Carolina Sabença, Rani de la Rivière, Paulo Barros, João Alexandre Cabral, Roberto Sargo, Luís Sousa, Maria de Lurdes Enes Dapkevicius, Filipe Silva, Filipa Lopes, Ana Carolina Abrantes, Madalena Vieira-Pinto, Manuela Caniça, Gilberto Igrejas, Carmen Torres, Patrícia Poeta

**Affiliations:** 1 MicroART-Antibiotic Resistance Team, Department of Veterinary Sciences, University of Trás-os-Montes and Alto Douro, 5000-801 Vila Real, Portugal; anacarolina@utad.pt; 2 Department of Genetics and Biotechnology, University of Trás-os-Montes and Alto Douro, 5000-801 Vila Real, Portugal; gigrejas@utad.pt; 3 Functional Genomics and Proteomics Unit, University of Trás-os-Montes and Alto Douro, 5000-801 Vila Real, Portugal; 4 Associated Laboratory for Green Chemistry, University NOVA of Lisbon, 1099-085 Caparica, Portugal; 5 National Reference Laboratory of Antibiotic Resistances and Healthcare Associated Infections, Department of Infectious Diseases, National Institute of Health, 1649-016 Lisbon, Portugal; raniriviere1404@gmail.com (R.d.l.R.); manuela.canica@insa.min-saude.pt (M.C.); 6 Centre for the Research and Technology of Agro-Environmental and Biological Sciences (CITAB), University of Trás-os-Montes e Alto Douro, 5000-801 Vila Real, Portugal; pbarros@utad.pt (P.B.); jcabral@utad.pt (J.A.C.); 7 Fluvial and Terrestrial Ecology Laboratory (LEFT), University of Trás-os-Montes and Alto Douro, 5000-801 Vila Real, Portugal; 8 CRAS-Center for the Recovery of Wild Animals, Veterinary Hospital, University of Trás-os-Montes and Alto Douro, 5000-801 Vila Real, Portugal; rsargo@utad.pt (R.S.); lsousa16@gmail.com (L.S.); fsilva@utad.pt (F.S.); 9 Faculty of Agricultural and Environmental Sciences, University of the Azores, 9700-042 Angra do Heroísmo, Portugal; maria.ln.dapkevicius@uac.pt; 10 Institute of Agricultural and Environmental Research and Technology (IITAA), University of the Azores, 9700-042 Angra do Heroísmo, Portugal; 11 CERAS, Wildlife Study and Rehabilitation Centre, Quercus ANCN, Rua Tenente Valadim 19, 6000-284 Castelo Branco, Portugal; ana.f.lopes@cm-lisboa.pt; 12 CECAV, Veterinary and Animal Research Centre, University of Trás-os-Montes and Alto Douro, 5000-801 Vila Real, Portugal; carolina.psca@gmail.com (A.C.A.); mmvpinto@utad.pt (M.V.-P.); 13 Department of Veterinary Sciences, University of Trás-os-Montes and Alto Douro, 5000-801 Vila Real, Portugal; 14 Associate Laboratory for Animal and Veterinary Science (AL4AnimalS), 5000-801 Vila Real, Portugal; 15 Centre for the Studies of Animal Science, Institute of Agrarian and Agri-Food Sciences and Technologies, University of Porto, 4051-401 Porto, Portugal; 16 Area of Biochemistry and Molecular Biology, OneHealth-UR Research Group, University of La Rioja, 26006 Logroño, Spain; carmen.torres@unirioja.es

**Keywords:** wildlife, environment, *Klebsiella* spp., *Raoultella* spp.

## Abstract

One of the significant challenges facing modern medicine is the rising rate of antibiotic resistance, which impacts public health, animal health, and environmental preservation. Evaluating antibiotic resistance in wildlife and their environments is crucial, as it offers essential insights into the dynamics of resistance patterns and promotes strategies for monitoring, prevention, and intervention. *Klebsiella* and *Raoultella* genera isolates were recovered from fecal samples of wild animals and environmental samples using media without antibiotic supplementation. Antibiograms were performed for 15 antibiotics to determine the phenotypic resistance profile in these isolates. Extended-spectrum β-lactamase (ESBL) production was tested by the double-disc synergy test, and one ESBL-producing *K. pneumoniae* isolate was screened by PCR and whole-genome sequencing. Biofilm production was analyzed using the microtiter plate method. A total of 23 *Klebsiella* spp. and 3 *Raoultella* spp. isolates were obtained from 312 fecal samples from wild animals, 9 *Klebsiella* spp. and 4 *Raoultella* spp. isolates were obtained from 18 river and stream water samples, and 4 *Klebsiella* spp. and 3 *Raoultella* spp. isolates from 48 soil samples. Regarding antibiotic resistance, only one isolate of *K. pneumoniae* from soil samples was an ESBL-producer and showed resistance to six antibiotics. This isolate harbored multiple β-lactams genes (*bla*_CTX-M-15_, *bla*_TEM-1_, *bla*_SHV-28_, and *bla*_OXA-1_), as well as genes of resistance to quinolones, sulfonamides, tetracycline, aminoglycosides, and chloramphenicol, and belonged to the lineage ST307. Most of the *Klebsiella* spp. and *Raoultella* spp. isolates were biofilm producers (except for one *Klebsiella* isolate), and 45.6% were weak biofilm producers, with the remaining being moderate to strong biofilm producers. We can conclude that antibiotic resistance is not widespread in these environment-associated isolates, which is a positive epidemiological outcome. However, identifying a single ESBL-*K. pneumoniae* isolate should serve as a warning of potential hotspots of resistance emergence.

## 1. Introduction

Enterobacteriaceae is a broad family of Gram-negative bacteria generally present in the gut of humans, animals, and other habitats like soil and water [1]. This family includes *Escherichia*, *Salmonella*, *Shigella*, *Klebsiella,* and *Raoultella* genera, among others, which have gained clinical and environmental importance. Several Enterobacteriaceae members are opportunistic pathogens that can cause infections ranging from mild gastroenteritis to severe urinary tract infections and even sepsis [2].

However, one of the significant challenges modern medicine faces is the increasing antimicrobial resistance (AMR) rates in public health, animal health, and environmental preservation. The possibility of Enterobacteriaceae acquiring antimicrobial resistance genes (ARGs) presents a severe challenge to human and veterinary medicine. [3]. The horizontal gene transfer of resistance traits among microorganisms contributes to the spread of resistant bacterial strains, particularly in the Enterobacteriaceae family. This significantly interferes with antibiotic treatments, often rendering them ineffective and leading to increased patient morbidity and mortality [4]. 

This situation is made even more complex by human, animal, and environmental health interconnectedness, as emphasized by the One Health approach [5]. Wildlife can act as reservoirs for antimicrobial-resistant bacteria, which may be passed on to domestic animals and humans, leading to a challenging cycle of resistance. Evaluating AMR in wildlife and their habitats is crucial, as it offers essential insights into the dynamics of resistance patterns and guides strategies for monitoring, prevention, and intervention [6]. 

Biofilms are complex microbial communities that attach to a surface and produce an extracellular matrix to protect themselves from environmental stressors, such as antibiotics [7]. Bacteria within biofilms have been reported to exhibit elevated AMR levels by up to 1000-fold compared to their planktonic counterparts due mainly to limited antibiotic diffusion, variable microenvironments, and reduced bacterial metabolic activity associated with the biofilm architecture [8]. Concerning *Klebsiella* spp. and *Raoultella* spp., biofilm formation not only enhances the survival of these microbes in environmental conditions but also allows the persistence and transmission of ARGs since horizontal gene transfer is more likely to occur because bacteria are much closer within the biofilm [9]. This resistance mechanism makes it difficult to control infections in human and veterinary medicine since such microorganisms have a higher chance of spreading resistant strains from wildlife and environmental sources, including soil and water, into clinical settings [10].

Thus, tackling AMR is vital for protecting public health, maintaining the health of our ecosystems, and ensuring that antibiotics remain effective for future generations. Recognizing the significance of AMR is vital to creating well-rounded policies and actions that address all facets of health and environmental care [11]. The primary aim of this study was to assess the levels of resistance and the ability to form biofilms in isolates of *Klebsiella* spp. and *Raoultella* spp. collected from various wild animals, rivers, streams, and soil samples. This analysis focused on the resistance profiles of these isolates, which were obtained from plates not supplemented with antibiotics, to gather information on the AMR levels and genetic traits of non-selected *Klebsiella* spp. and *Raoultella* spp. isolates found in wildlife and their surrounding environments.

## 2. Materials and Methods

### 2.1. Study Area Selection

**Figure 1:**
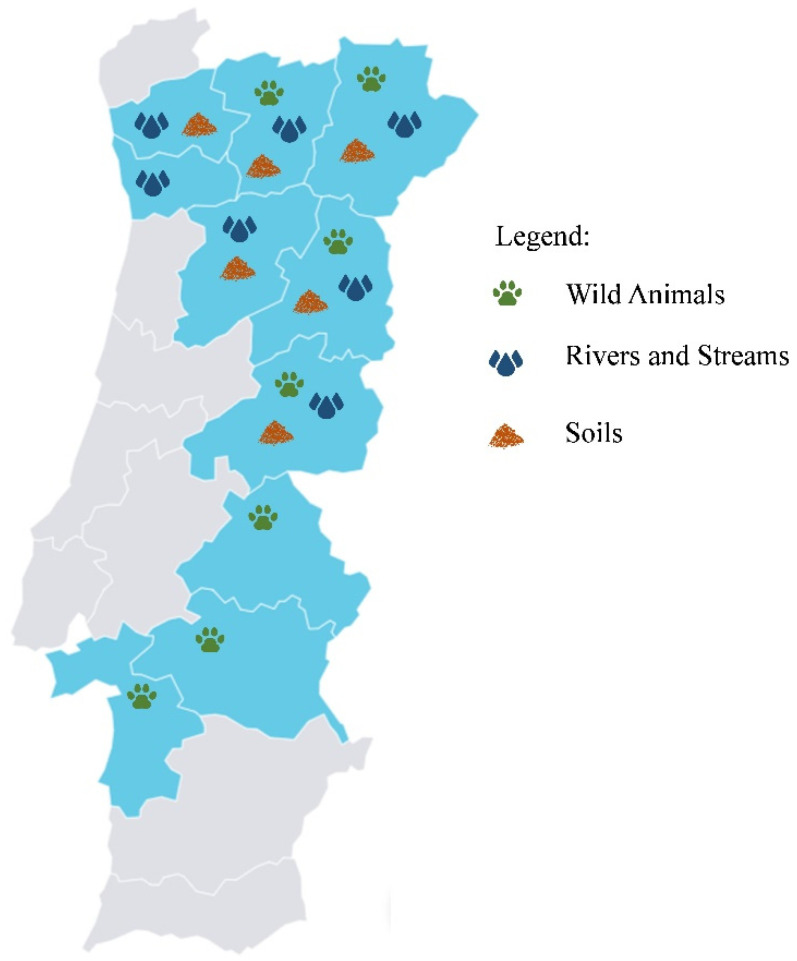
Map of Portugal showing the area of study where wild animals, rivers and streams, and soils were sampled.

The present study was performed in a total of ten districts of Portugal. Wildlife samples were collected in Bragança, Castelo Branco, Évora, Guarda, Portalegre, Setúbal, and Vila Real. Rivers and stream water samples were collected in Braga, Bragança, Castelo Branco, Guarda, Porto, Vila Real, and Viseu. Soil samples were collected in Braga, Bragança, Guarda, Castelo Branco, Vila Real, and Viseu (Figure 1). All water and soil locations were selected in an attempt to approximate the locations where the wild animals were recovered. In the districts of Setúbal, Évora, and Portalegre, water or soil samples could not be collected due to the extended distance from the laboratory. 

### 2.2. Sampling Techniques

#### 2.2.1. Wildlife Feces Sampling

**Figure 2:**
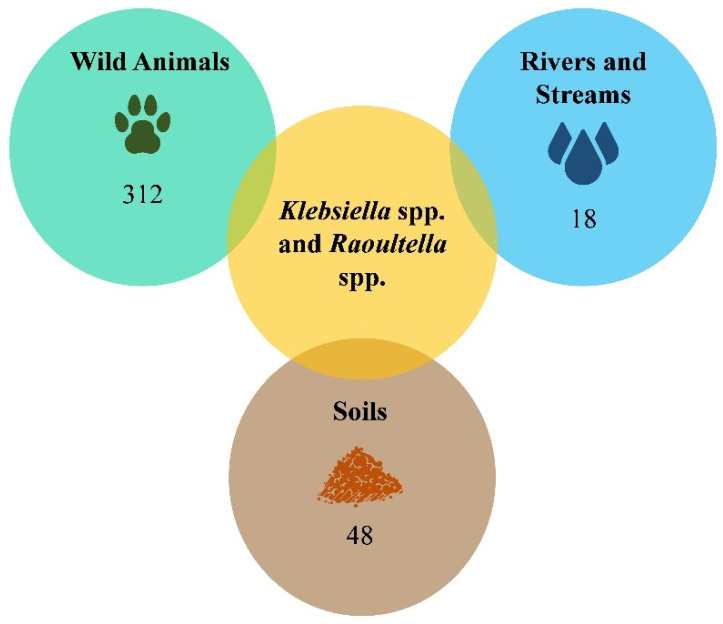
Total number of wild animals, water, and soil samples screened for *Klebsiella* spp. and *Raoultella* spp.

The wild animals reported in this study were divided into three groups according to the sampling method: (1) wild animals from recovery centers; (2) bats; and (3) wild boars. A total of 312 wild animals were sampled for this study (Figure 2).

Regarding the samples obtained by the recovery center, a total of 42 fecal samples were collected (one sample per animal) between September 2021 and December 2021 by the CRAS (Centro de Recuperação de Animais Selvagens) institution and CERAS (Centro de Estudos e Recuperação de Animais Selvagens) institution. The collection was made as soon as the animals made feces on their own in the first days of hospitalization, using a spatula and placing the fresh feces directly into the sterilized shipping container. Concerning samples obtained from bats, a trap specifically designed to increase the trapping efficiency of bats was used to capture the animals from dusk to dawn. A total of 95 animals (one sample per animal) were captured between May 2022 and September 2022 and kept in individual cotton bags until they were processed (sexed, aged, weighed, and measured for forearm length), after which fecal samples were subsequently collected in sterile tubes [12]. Bat capture and handling adhered to applicable guidelines and regulations and received approval from the Ethical Committee at the ICNF (Instituto da Conservação da Natureza e das Florestas) (license Nº 026/2022/CAPT). Respecting wild boars, a total of 175 animals were sampled (one sample per animal) between January 2020 and February 2022. Their feces recovery was completed during hunts of wild boars, and all samples were collected from wild boars that were legally hunted. This study did not involve the deliberate killing of animals. Within a short time after death, during the evisceration in the collection point after each driven hunt, fecal samples (~25 g) were collected from the posterior part of the large intestine. All samples were kept at 4 °C and transported to the lab within 12 h.

#### 2.2.2. River and Stream Water Sampling

A total of 18 water samples were collected (one sample per location) between July 2022 and April 2023 (Figure 2). Water samples from rivers and streams were aseptically collected in sterile 500 mL bottles by immersing them directly into the water’s surface [12]. The bottles were correctly labeled and transported on ice to the laboratory for analysis.

#### 2.2.3. Soil Sampling

The soil samples were collected from 16 locations between July 2022 and April 2023. Three meter-squared plots were randomly selected at each location. Topsoil was collected using a small trowel by randomly selecting ten points in each plot, yielding a composite sample representative of the meter-squared plot. The soil samples were then placed into sterile plastic bags labeled with the site name, refrigerated, and stored at 4 °C as soon as possible [13,14]. Three samples were collected from each of the 16 locations, resulting in a total of n = 48 soil samples (Figure 2).

### 2.3. Laboratory Methods for Isolation and Identification

#### 2.3.1. Bacterial Isolation from Wildlife

The methodology to isolate *Klebsiella* spp. and *Raoultella* spp. from fecal samples of wildlife was the same as previously described by Sabença et al. [15]. HiCrome *Klebsiella* Selective Agar Base (HiMedia Laboratories, Einhausen, Germany) and Chromogenic Coliform Agar (Oxoid, Cheshire, England), both media without supplementation were used to isolate the bacterial isolates. To differentiate *Klebsiella* spp. and *Raoultella* spp. isolates, MALDI-TOF (Bruker, Billerica, MA, USA) was used to identify them and were then kept at −20 °C for further studies.

#### 2.3.2. Bacterial Isolation from Rivers and Streams

The isolation of *Klebsiella* spp. and *Raoultella* spp. from water sources was made by membrane filtration. For all the samples, three volumes of 100 mL were filtered through a 0.45 µm poresized filter (cellulose nitrate membranes, Whatman Laboratory Division, Maidstone, England) using a water pump [12]. These membranes were aseptically cut, placed in tubes with 5 mL of Brain Heart Infusion broth (BHIb) (Liofilchem, Roseto degli Abruzzi, Italy), and maintained at 37 °C for 24 h. After incubation, the procedure was the same as in Sabença et al. [15].

#### 2.3.3. Bacterial Isolation from Soils

For isolation, soil plastic bags were extensively shaken to homogenize each plot’s ten collection points. Then, 1 g of soil from each plot was transferred to tubes with 5 mL of BHIb and maintained at 37 °C for 24 h. After incubation, the procedure was the same as in Sabença et al. [15].

### 2.4. Antibiotic Susceptibility Testing

The phenotypic resistance characterization of the *Klebsiella* spp. and *Raoultella* spp. isolates was performed by the Kirby–Bauer disk diffusion method by following EUCAST standards (2022) [16], except for ceftazidime, cefotaxime, tetracycline, and nalidixic acid, for which CLSI standards (2021) were followed [17]. *Escherichia coli* ATCC® 25922 was used as a control strain. We used the double-disc synergy test to identify potential isolates that produced ESBL enzymes. A total of 15 antibiotics were used in the susceptibility testing of *Klebsiella* spp. and *Raoultella* spp. isolates (in μg/disk): amoxicillin–clavulanic acid (AUG, 20-10), cefoxitin (FOX, 30), cefotaxime (CTX, 5), ceftazidime (CAZ, 10), cefepime (FEP, 30), aztreonam (ATM, 30), imipenem (IMI, 10), ertapenem (ERT, 10), meropenem (MEM, 10), amikacin (AMI, 30), gentamicin (GEN, 10), ciprofloxacin (CIP, 5), trimethoprim–sulfamethoxazole (SXT, 1.25/23.75), chloramphenicol (CHL, 30), and tetracycline (TET, 30). *Klebsiella* spp. and *Raoultella* spp. isolates were selected for further studies (one isolate per sample of wildlife, water, and soil). Following this criterion, a collection of 36 *Klebsiella* spp. and 10 *Raoultella* spp. isolates was characterized in this study. Isolates resistant to three or more antibiotic classes were considered multidrug resistant (MDR).

### 2.5. Detection of Resistance Genes by PCR

DNA extraction from one *K. pneumoniae* isolate was performed using the boiling method; briefly, one colony of an overnight culture was suspended in 1 mL of MilliQ water, and later, it was boiled for 8 min to break down the cell wall and centrifuged at 12,000 rpm for 2 min to remove the pellet. DNA concentration was checked using the Nanodrop spectrophotometer [18].

This *K. pneumoniae* isolate was then screened for multiple resistance genes according to its resistance profile. By PCR, we analyzed the presence of antimicrobial resistance genes: β-lactams (*bla*_CTX-M-group 1_), TET (*tet*A, and *tet*B), SXT (*sul*1, *sul*2, and *sul*3), and CIP (*qnr*S, *qnr*A, and *qnr*B) [18,19]. The presence of class 1 integrons was determined by PCR (*int*I1) [20]. Positive and negative controls from the University of La Rioja (Spain) strain collection were included in all PCR assays. PCR reactions were performed using gene-specific primers, and the details of primer sequences, annealing temperatures, amplicon size, and cycling conditions can be seen in Table S1. Finally, the amplified products were visualized on 1% agarose gels with green safe incorporated and confirmed based on their expected amplicon sizes.

### 2.6. Whole-Genome Sequencing

Whole-genome sequencing (WGS) was performed in one ESBL-producing *K. pneumoniae* strain isolated from a soil sample to confirm the identity of the PCR-amplified resistance genes and rule out potential nonspecific amplification. Initially, the strain was seeded in the differential medium, MacConkey, and isolated in nutrient agar. The genomic DNA was extracted using the Magna Pure 96 system (Roche, Basel, Switzerland), according to the manufacturer’s protocol, and DNA concentration was measured using a Qubit^TM^ 4 fluorometer (Thermo Scientific, Waltham, MA, USA). Sequencing libraries were prepared using the Nextera XT library preparation kit (Illumina, San Diego, CA, USA) and sequenced using Illumina MiSeq with 150 bp paired-end reads. Raw reads were then submitted to bioinformatics analysis with multiple tools to process WGS data. De novo assembly, species confirmation, sequence type, and raw data quality control were performed using INNUca (v 4.2.2-02) (https://github.com/B-UMMI/INNUca accessed on 23 September 2024). The assessment of read quality, trimming, and estimated genome completeness were performed using FastQC (v 0.11.5) (http://www.bioinformatics.babraham.ac.uk/projects/fastqc/; accessed on 23 September 2024), Trimmomatic (v 0.38) [21], and BUSCO (v 5.5.0_cv1) (https://gitlab.com/ezlab/busco#how-to-cite-busco; accessed on 23 September 2024), respectively. The identification of the species was confirmed by calculating average nucleotide identity (ANI) using FastANI (v 1.33) [22], where the query genome is compared against the complete assembled reference genomes downloaded from the NCBI Genbank database (https://www.ncbi.nlm.nih.gov/datasets/genome/?taxon=570; accessed on 23 September 2024). For ARG detection, abriTAMR (v 1.0.14) and ABRicate (v 1.0.1) were used (http://github.com/tseemann/abricate; accessed on 23 September 2024) [23]. In ABRicate, the following public databases were used: argannot, resfinder, ncbi, card, plasmidfinder, and vfdb.

### 2.7. Biofilm Formation and Quantification Assays

The biofilm formation capacity of all bacterial isolates was evaluated using a microtiter plate-based assay, following previously described methods by Sabença et al. [24]. 

## 3. Results

### 3.1. Isolation and Identification of Klebsiella spp. and Raoultella spp.

#### 3.1.1. Wild Animals

A total of 23 *Klebsiella* spp. isolates were recovered from 23 of the 312 fecal samples collected (7.4%), and 3 *Raoultella* spp. isolates were recovered from 3 of the 312 fecal samples collected (0.96%) using media non-supplemented with antibiotics (Table 1). The 23 animals in which *Klebsiella* spp. isolates could be recovered were from: (A) recovery centers: *Erinaceus europaeus* (n = 4) and *Tyto alba* (n = 1); (B) from bats: *Nyctalus leisleri* (n = 2), *Rhinolophus mehelyi* (n = 1), *Plecotus auritus* (n = 1), and *Plecotus austriacus* (n = 4); (C) from wild boars: *Sus scrofa* (n = 10). The three animals in which *Raoultella* spp. isolates could be recovered were as follows: (A) bats: *Tadaria teniotis* (n = 1), and *Pipistrellus pipistrellus* (n = 1); (B) wild boars: *Sus scrofa* (n = 1).

Among the *Klebsiella* spp. isolates, *K. pneumoniae* (n = 11), *K. oxytoca* (n = 7), and *K. variicola* (n = 5) were obtained. Among the *Raoultella* spp. isolates, *R. planticola* (n = 1) and *R. ornithinolytica* (n = 2) were identified.

#### 3.1.2. Rivers and Streams 

A total of 9 *Klebsiella* spp. isolates were recovered from 9 of the 18 water samples collected (50%), and 4 *Raoultella* spp. isolates were recovered from 4 of the 18 water samples collected (22.2%) using media non-supplemented with antibiotics (Table 1).

Among the *Klebsiella* spp. isolates, *K. pneumoniae* (n = 8) and *K. oxytoca* (n = 1) were identified. Among the *Raoultella* spp. isolates, only *R. planticola* was detected.

#### 3.1.3. Soils

A total of 4 *Klebsiella* spp. isolates were recovered from 4 of the 48 soil samples collected (8.3%), and 3 *Raoultella* spp. isolates were recovered from 3 of the 48 soil samples collected (6.3%), using media non-supplemented with antibiotics (Table 1).

Among the *Klebsiella* spp. isolates, *K. pneumoniae* (n = 3) and *K. oxytoca* (n = 1) were identified. Among the *Raoultella* spp. isolates, only *R. planticola* (n = 3) was detected.

### 3.2. Antibiotic Resistance Profiles 

Only one isolate of *K. pneumoniae*, isolated among the soil samples, showed resistance to six antibiotics (amoxicillin–clavulanic acid, ceftazidime, cefotaxime, ciprofloxacin, trimethoprim–sulfamethoxazole, and tetracycline), belonging to four different antibiotic classes. Since this isolate was resistant to more than three antibiotic classes, it is considered an MDR isolate. This isolate was also positive for ESBL production. In contrast, all other isolates of *Klebsiella* spp. and *Raoultella* spp. showed susceptibility for all antibiotics tested (Table 2). 

### 3.3. Genomic Characterization by PCR and Whole Genome Sequencing of ESBL-Producing Isolate

The PCR and WGS were performed in the unique ESBL-producing *K. pneumoniae* isolate detected in this study. Initially, by PCR, we detected *bla*_CTX-M-group1_, *tet*A, *sul*2, and *qnr*B resistance genes, and *Int*I1 integrase, accountable for the movement of antibiotic resistance cassettes within integrons (Table 2). WGS confirmed the presence of these genes detected by PCR and additionally revealed more information. The ESBL-producing *K. pneumoniae* isolate belonged to the ST307 sequence type and harbored multiple β-lactams genes, such as *bla*_CTX-M-15_, *bla*_TEM-1_, *bla*_SHV-28_, and *bla*_OXA-1_. Additional resistance genes were detected, which included *qnr*B1, *oqx*A, *oqx*B19, and *aac*(6′)-Ibcr, conferring resistance to quinolones, *sul*2, and *dfr*A14, regarding resistance to sulfonamides and trimethoprim, and *tet*A, concerning resistance to tetracycline. We also detected *aph*(6)-Id, *aph*(3″)-Ib, and *acr*D genes that confer resistance to aminoglycosides, and *cat*B3 that confer resistance to chloramphenicol; no resistance to these classes was detected in this study (Table 3).

We also identified three different plasmids: IncFIB(K), IncFII, and ColRNAI. Concerning virulence genes, we detected *ybt*Q and *ybt*P genes. Several heavy metal resistance genes, including the silver resistance genes (*sil*), the plasmid-borne copper resistance system (*pco*) gene cluster, and the *ars* operon genes conferring resistance to arsenic, were identified (Table 3).

### 3.4. Biofilm Formation Assay

**Figure 3:**
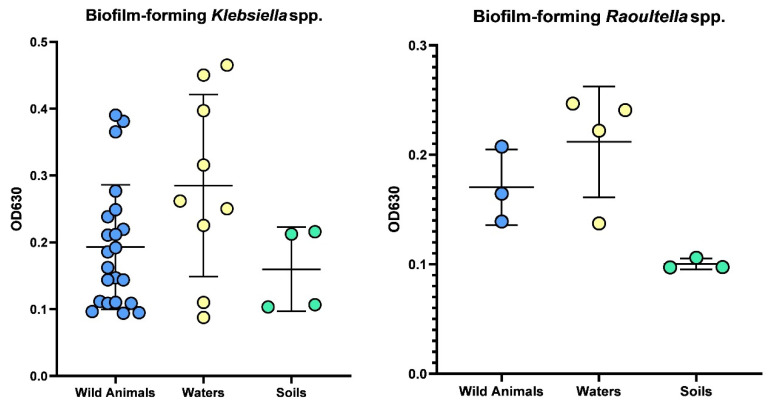
Comparison of biofilm produced between *Klebsiella* spp. and *Raoultella* spp. isolates among wildlife, water, and soil samples.

Among all *Klebsiella* spp. and *Raoultella* spp. isolates analyzed, only one *Klebsiella* spp. isolate did not produce biofilm. Of the 35 *Klebsiella* spp. isolates that produced biofilm, 15 were weak biofilm producers, 13 were moderate producers, and 7 were strong producers. Among the 10 *Raoultella* spp. isolates, 6 were weak biofilm producers, 4 were moderate, and none were strong producers (Table 2 and Figure 3).

## 4. Discussion

This study was carried out to assess the antimicrobial resistance and the biofilm formation capacities in *Klebsiella* spp. and *Raoultella* spp. isolates from wildlife and their environments, using a cultivation strategy without antibiotic selection to evaluate the actual AMR prevalence among non-selected isolates of the intestinal tract of the animals, rivers and streams, and soils. The data are important for defining effective strategies to control AMR and monitor its progress over time. Given the nearness of animals and humans in several rural and urban settings, monitoring AMR in wildlife populations and their environments is crucial to prevent the dissemination of MDR bacteria in both directions. The formation of biofilms by *Klebsiella* spp. and *Raoultella* spp. isolates is a grand concern, as it can disseminate antibiotic resistance. Additionally, the majority of our isolates showed antimicrobial susceptibility; this does not impede their capacity to form biofilms since it is a mechanism that provides them protection from environmental stresses, such as the increase in antibiotic levels in their surroundings. 

In this work, we isolated *Klebsiella* spp. in approximately 7% of the wild animals, 50% of the rivers and streams water samples, and 8% of the soil samples. We also isolated *Raoultella* spp. in approximately 1% of wild animals, 22% of river and stream water samples, and 6% of soil samples. Multiple studies have also reported the successful isolation of both bacterial species among wildlife [25,26], water samples [27,28], and soil [29,30]. Our findings suggest that wild animals, rivers and streams water, and soils can act as reservoirs for *Klebsiella* spp. and *Raoultella* spp. The high isolation rate observed in water samples suggests that aquatic environments may be essential niches for these bacteria. This is particularly relevant as water can serve as a transmission route to other animals, humans, or the environment [31].

Wildlife can act as reservoirs for antibiotic-resistant bacteria and resistance genes due to direct or indirect exposure to anthropogenic sources, such as agricultural runoff, untreated wastewater, or improperly managed antimicrobial use in veterinary and human medicine [32]. These exposures enable the horizontal transfer of resistance determinants from human-associated bacteria to those inhabiting natural ecosystems.

Wildlife helps monitor the emergence and dissemination of antibiotic resistance (AR) by serving as sentinels, particularly in areas with limited human access [32]. For example, birds, which are highly mobile and capable of spanning large geographic areas, can transport resistant bacteria between ecosystems, acting as vectors of resistance [33,34]. Similarly, aquatic animals and mammals near polluted water sources can provide early warning signals of environmental contamination with antimicrobial agents or resistant organisms [35]. This bridging role of animals underscores the interconnectedness of human, animal, and environmental health within the framework of the One Health approach. Investigating the presence of AR in wildlife not only highlights the movement of resistance genes between human-dominated and natural ecosystems but also identifies potential risks to public health, agriculture, and biodiversity conservation.

Generally, in articles on antimicrobial resistance, when we come across the topic of resistance to antibiotics, we are presented with a varied list of antibiotics to which the isolates are resistant, and very few are given as antimicrobial susceptible. In this work, we came across the opposite, as only one MDR and ESBL-producing *K. pneumoniae* was isolated from soil samples. This ESBL-producing isolate showed resistance to amoxicillin–clavulanic acid, ceftazidime, cefotaxime, ciprofloxacin, trimethoprim–sulfamethoxazole, and tetracycline. Recently, a study conducted in poultry farms isolated five MDR *K. pneumoniae* from water and soil samples and three of them were ESBL producers. Concerning the resistance patterns, the authors of this study reported resistance to amoxicillin–clavulanic acid, cefuroxime, cefoxitin, cefotaxime, ceftazidime, ceftriaxone, cefepime, aztreonam, trimethoprim-sulfamethoxazole, ciprofloxacin, and nitrofurantoin among *K. pneumoniae* isolates [36]. A broader resistance pattern was observed in poultry farms and may reflect a selection for higher rates of MDR bacteria by antibiotic usage in agricultural settings. These contrasts between findings in natural environments versus agricultural sites suggest that environmental isolates may harbor fewer resistance traits due to reduced antibiotic pressure in non-intensive settings. These findings raise interesting questions about the selective pressure for developing AMR across various environments. Although MDR and ESBL-producing *K. pneumoniae* are frequent in antibiotic-exposed agricultural settings, low prevalence in our environmental samples suggests that there may be little dissemination of resistance genes in settings with lower antibiotic exposure. This disparity underlines the role of agricultural use practices in developing and maintaining resistance, pointing to the role of environment-specific pressures in shaping resistance profiles.

Multiple β-lactams genes were detected in our soil ESBL-producing *K. pneumoniae* isolate, such as *bla*_CTX-M-15_, *bla*_TEM-1_, *bla*_SHV-28_, and *bla*_OXA-1_, indicating a solid β-lactam resistance profile. Similar results were obtained by one study conducted on soil samples from a landfill, a preservation area, and a farm [37]; spread among the different regions were *bla*_SHV_, *bla*_TEM-116_, and *bla*_OXA-1_ genes, but the *bla*_CTX-M-14_ variant was only detected in the landfill and farm soil samples [37]. The isolation of these soil strains with multiple ARGs highlights the potential environmental variations in resistance gene prevalence and suggests that specific habitats may drive the selection of distinct β-lactamase variants. The presence of quinolone resistance genes (*qnr*B1, *oqx*A, *oqx*B19, and *aac*(6′)-Ib-cr), sulfonamide and trimethoprim resistance genes (*sul*2 and *dfr*A14), and the tetracycline resistance gene (*tet*A), underscore the multidrug-resistant profile of this isolate. Similar results were reported by Wang et al., who described the profile and dynamics of the antibiotic resistome and pathogens across four compartments of a soil–root continuum (the unplanted soil, rhizosphere, episphere, and endosphere). Most of the resistance genes they detected were present in the four compartments, mostly encoding resistance to tetracyclines, MLS_B_ (macrolide–lincosamide–streptogramin B), glycopeptides, and fluoroquinolones. Notably, sulfonamide and trimethoprim resistance genes were rare in Wang’s study, contrasting with the prevalence of *sul*2 and *dfr*A14 in our findings [38]. This suggests environmental factors unique to our samples, possibly linked to local antibiotic use or contamination sources. We also detected the *aph*(6)-Id, *aph*(3″)-Ib, *acr*D genes that confer resistance to aminoglycosides, and *cat*B3, which confer resistance to chloramphenicol. However, susceptibility testing did not confirm phenotypic expression, suggesting these genes may be silenced under current conditions [39]. The *Int*I1 integrase gene was also detected by PCR, a key element in horizontal gene transfer, further implying that our isolate can acquire and disseminate resistance traits, which aligns with reports of integron presence in environmental bacteria as a mechanism for resistance spread [40]. Meanwhile, the rest of the *Klebsiella* spp. and *Raoultella* spp. isolates were susceptible to all antibiotics tested. Håkonsholm et al. found a similarly low prevalence of antibiotic resistance among *Klebsiella* spp. and *Raoultella* spp. isolated from marine bivalves in Norway. Specifically, the authors reported that only a few *K. pneumoniae* isolates showed resistance to multiple antibiotics, with three being classified as MDR. One *K. pneumoniae* isolate carried an ESBL gene (*bla*_CTX-M-3_) and showed resistance to cefotaxime. Still, most of the isolates in this study were susceptible to most antibiotics tested, similar to our results [41]. It does not detract from the overall concern about antibiotic resistance across environmental and animal-associated isolates. Several studies described resistance genes in an array of ecological niches, including birds [42], soil [43], and water [43], pointing to antibiotic resistance as an ecologically diffused problem. These results suggest that, although some particular isolates or settings may show low resistance, environmental and wildlife reservoirs remain essential contributors to the dissemination and persistence of resistance genes across ecosystems. 

Regarding biofilm production, nearly all *Klebsiella* spp. isolates (35 out of 36) produced biofilm, with varying levels of biofilm-forming capacity (15 weak, 13 moderate, and 7 strong producers). *Raoultella* spp. isolates could also produce biofilms, though none were strong producers, with six weak and four moderate producers. The biofilm-forming capabilities of these bacteria suggest that they have mechanisms for long-term persistence in various environments [44]. This could enable them to survive in harsh conditions, increasing their potential to spread to other animals or humans and possibly acquire resistance genes over time [45]. Biofilm-associated bacteria are significantly more resistant to antibiotics than their planktonic counterparts. Resistance is enhanced because the biofilm matrix may prevent effective antibiotic penetration, it can produce nutrient-limited zones that retard bacterial metabolism, and it may concentrate resistance genes within the biofilm community by facilitating gene exchange. The capability of *Klebsiella* and *Raoultella* species to form biofilms in both the environment and animal hosts raises an increased risk for the dissemination and persistence of AMR bacteria. These biofilms act as a reservoir for multidrug resistance, wherein resistant strains may spread from environmental sources into clinical settings. Thus, our study’s high prevalence of biofilm-forming isolates points to a critical mechanism underpinning persistence in environmental reservoirs and the broader dissemination of AMR.

This study’s observed antibiotic resistance patterns highlight the potential public health and environmental risks associated with antibiotic-resistant bacteria in wildlife and environmental samples. The detection of an MDR *K. pneumoniae* isolate in soil, which exhibited resistance to multiple antibiotics and carried multiple resistance genes, underscores the environmental reservoirs for resistance and the potential for horizontal gene transfer. While most wildlife isolates were susceptible to antibiotics, their role as sentinels and potential reservoirs of resistance emphasizes the need for surveillance, as they can bridge the gap between human-influenced environments and natural ecosystems. Environmental compartments such as rivers, streams, and soil act as key sources for disseminating antibiotic-resistant bacteria, which can persist due to biofilm formation, further exacerbating the spread of resistance. These findings reinforce the need for a One Health approach to AMR surveillance, integrating human, animal, and environmental health to mitigate the risks of resistance spread.

Nevertheless, one limitation of this study is its sample size, which includes a relatively small number of isolates. Besides the existence of a single ESBL-producing isolate being a positive result in this context, it may not reflect the general population since no antibiotic was used to select ESBL isolates. Also, more extensive surveillance would be necessary to confirm that antibiotic resistance remains low. Because resistance levels can change according to geographic location, healthcare practices, and time frame, this finding is positive for this specific moment, and continuous monitoring is necessary to ensure the resistance levels do not elevate. Even with just one resistant isolate, this ESBL producer could be a source for future transmission. ESBL-producing bacteria have the potential to spread fast if infection control measures are not robust.

## 5. Conclusions

Conclusively, this research provides insight into the degree of AMR and biofilm-forming potentials among *Klebsiella* spp. and *Raoultella* spp. from wildlife and environmental samples. The results show that although the isolates were mainly susceptible to antibiotics, the ability of these biofilm-forming bacteria to survive in different environments may suggest a role in promoting AMR dissemination over extended periods. Single isolation of MDR *K. pneumoniae* ESBL-producing from the soil is a crucial reminder of the potential reservoirs of resistance. Although resistance was not widespread, the biofilm potential and presence of antibiotic-resistance genes demonstrated that ongoing surveillance is essential. Therefore, strict vigilance is required in rural and urban areas where there are higher chances of close contact between animals and humans to avoid the spread of MDR strains. This information may then be used to further understand AMR dynamics in future studies (using larger sample sizes and more comprehensive surveillance efforts) and consequently assist in maintaining a low sustained prevalence of resistance.

## Figures and Tables

**Table 1:** Isolation of *Klebsiella* spp. and *Raoultella* spp. from wild animals and environment samples.

	*Klebsiella pneumoniae* (n = 22)	*Klebsiella oxytoca* (n = 9)	*Klebsiella variicola* (n = 5)	Total *Klebsiella* spp.	*Raoultella planticola* (n = 8)	*Raoultella ornithinolytica* (n = 2)	Total *Raoultella* spp.
**Wild Animals**	11	7	5	23	1	2	3
**Rivers and streams**	8	1	0	9	4	0	4
**Soils**	3	1	0	4	3	0	3

**Table 2:** Characteristics of microbial isolates from wildlife, water, and soil samples.

	Isolate	Animal (ID)	Species	Resistance Profile	Genes Detected by PCR	ESBL Producer	Biofilm Type
Wild Animals	NL1	*Nyctalus leisleri* (nº10)	*Klebsiella oxytoca*	Susceptible to all antibiotics	-	N	Weak
	TT1	*Tadaria teniotis* (nº34)	*Raoultella planticola*	Susceptible to all antibiotics	-	N	Weak
	PP9	*Pipistrellus pipistrellus* (nº41)	*Raoultella ornithinolytica*	Susceptible to all antibiotics	-	N	Moderate
	RM2	*Rhinolophus mehelyi* (nº53)	*Klebsiella pneumoniae*	Susceptible to all antibiotics	-	N	Moderate
	PA1	*Plecotus auritus* (nº77)	*Klebsiella oxytoca*	Susceptible to all antibiotics	-	N	Weak
	PAus2	*Plecotus austriacus* (nº71)	*Klebsiella oxytoca*	Susceptible to all antibiotics	-	N	Weak
	PAus5	*Plecotus austriacus* (nº73)	*Klebsiella oxytoca*	Susceptible to all antibiotics	-	N	Strong
	PAus9	*Plecotus austriacus* (nº78)	*Klebsiella oxytoca*	Susceptible to all antibiotics	-	N	Moderate
	PAus14	*Plecotus austriacus* (nº79)	*Klebsiella oxytoca*	Susceptible to all antibiotics	-	N	Moderate
	NL8	*Nyctalus leisleri* (nº4)	*Klebsiella oxytoca*	Susceptible to all antibiotics	-	N	Weak
	TA5	*Tyto alba* (5120/N904)	*Klebsiella pneumoniae*	Susceptible to all antibiotics	-	N	Moderate
	OC7	*Erinaceus europaeus* (5049/M521)	*Klebsiella pneumoniae*	Susceptible to all antibiotics	-	N	Moderate
	OC16	*Erinaceus europaeus* (4927/M511)	*Klebsiella pneumoniae*	Susceptible to all antibiotics	-	N	Moderate
	OC24	*Erinaceus europaeus* (4906/M508)	*Klebsiella pneumoniae*	Susceptible to all antibiotics	-	N	Weak
	OC37	*Erinaceus europaeus* (4908/M509)	*Klebsiella pneumoniae*	Susceptible to all antibiotics	-	N	Weak
	J1	*Sus scrofa* (3 S. Seb)	*Klebsiella pneumoniae*	Susceptible to all antibiotics	-	N	Moderate
	J45	*Sus scrofa* (HVE86)	*Klebsiella pneumoniae*	Susceptible to all antibiotics	-	N	Strong
	J53	*Sus scrofa* (HVE80)	*Klebsiella pneumoniae*	Susceptible to all antibiotics	-	N	Strong
	J90	*Sus scrofa* (HVE 39)	*Klebsiella variicola*	Susceptible to all antibiotics	-	N	Weak
	J93	*Sus scrofa* (HVE 2)	*Klebsiella variicola*	Susceptible to all antibiotics	-	N	Weak
	J99	*Sus scrofa* (HVE 20)	*Klebsiella variicola*	Susceptible to all antibiotics	-	N	-
	J100	*Sus scrofa* (HVE 48)	*Klebsiella pneumoniae*	Susceptible to all antibiotics	-	N	Weak
	J105	*Sus scrofa* (HVE 4)	*Klebsiella pneumoniae*	Susceptible to all antibiotics	-	N	Moderate
	J115	*Sus scrofa* (HVE 29)	*Klebsiella variicola*	Susceptible to all antibiotics	-	N	Weak
	J123	*Sus scrofa* (HVE 22)	*Klebsiella variicola*	Susceptible to all antibiotics	-	N	Weak
	J142	*Sus scrofa* (HEV 105)	*Raoultella ornithinolytica*	Susceptible to all antibiotics	-	N	Weak
River and Streams Waters	W4	NA	*Klebsiella oxytoca*	Susceptible to all antibiotics	-	N	Weak
	W8	NA	*Raoultella planticola*	Susceptible to all antibiotics	-	N	Moderate
	W9	NA	*Raoultella ornithinolytica*	Susceptible to all antibiotics	-	N	Weak
	W19	NA	*Klebsiella pneumoniae*	Susceptible to all antibiotics	-	N	Moderate
	W25	NA	*Klebsiella oxytoca*	Susceptible to all antibiotics	-	N	Moderate
	W37	NA	*Klebsiella oxytoca*	Susceptible to all antibiotics	-	N	Strong
	W41	NA	*Klebsiella oxytoca*	Susceptible to all antibiotics	-	N	Moderate
	W45	NA	*Klebsiella oxytoca*	Susceptible to all antibiotics	-	N	Strong
	W57	NA	*Klebsiella oxytoca*	Susceptible to all antibiotics	-	N	Strong
	W60	NA	*Klebsiella oxytoca*	Susceptible to all antibiotics	-	N	Strong
	W65	NA	*Klebsiella pneumoniae*	Susceptible to all antibiotics	-	N	Moderate
	W84	NA	*Klebsiella pneumoniae*	Susceptible to all antibiotics	-	N	Moderate
	W95	NA	*Klebsiella pneumoniae*	Susceptible to all antibiotics	-	N	Weak
Soils	S7	NA	*Klebsiella pneumoniae*	AUG CAZ CTX CIP SXT TET	*bla*_CTX-M-group 1_; *tet*A; *int*I1; *sul*2; *qnr*B	P	Moderate
	S17K	NA	*Klebsiella pneumoniae*	Susceptible to all antibiotics	-	N	Weak
	S27	NA	*Klebsiella pneumoniae*	Susceptible to all antibiotics	-	N	Weak
	S33	NA	*Klebsiella pneumoniae*	Susceptible to all antibiotics	-	N	Weak
	S45	NA	*Klebsiella pneumoniae*	Susceptible to all antibiotics	-	N	Weak
	S51	NA	*Klebsiella variicola*	Susceptible to all antibiotics	-	N	Weak
	S78	NA	*Klebsiella variicola*	Susceptible to all antibiotics	-	N	Moderate

NA: not applicable; AUG: amoxicillin–clavulanic acid; CAZ: ceftazidime; CTX: cefotaxime; CIP: ciprofloxacin; SXT: trimethoprim–sulfamethoxazole; TET: tetracycline; ESBL: extended-spectrum β-lactamase; N: negative; P: positive.

**Table 3:** Genomic Features of the ESBL-producing *K. pneumoniae* isolate.

Isolate	MLST	β-lactamase Genes	Other Resistance Genes	Plasmids	Virulence Genes	Heavy Metal Resistance Genes
S7	ST307	*bla*_CTX-M-15_, *bla*_TEM-1_, *bla*_SHV-28_, *bla*_OXA-1_	*qnr*B1, *oqx*A, *oqx*B19, *aac*(6′)-Ib-cr, *aph*(6)-Id, *aph*(3″)-Ib, *acr*D, *tet*A, *sul*2, *dfr*A14, *cat*B3	IncFIB(K), IncFII, ColRNAI	*ybt*Q, *ybt*P	*sil*E, *sil*S, *sil*R, *sil*C, *sil*F, *sil*B, *sil*A, *sil*P, *pco*A, *pco*B, *pco*C, *pco*D, *pco*R, *pco*S, *pco*E, *ars*C, *ars*B, *ars*A, *ars*D, *ars*R

## Data Availability

The authors confirm that the data supporting the findings of this study are available within the article.

## Supplementary Materials

The following supporting information can be downloaded at: https://www.mdpi.com/article/10.3390/pathogens14010099/s1, Table S1: Primers sequences and PCR conditions carried out in this study. References [46,47] are cited in the supplementary materials.
