# Supplementary material for: Assessment of Antibiotic Resistance Among Isolates of Klebsiella spp. and Raoultella spp. in Wildlife and Their Environment from Portugal: A Positive Epidemiologic Outcome
Source: Pathogens. 2025 Jan 20;14(1):99. doi: 10.3390/pathogens14010099 (PMC11768959; doi:10.3390/pathogens14010099)
Supplement: Supplementary file 1 [file pathogens-14-00099-s001.zip › pathogens-3422139-supplementary.pdf]

Table S1: Primers sequences and PCR conditions carried out in this study.

Target Gene	Primer Sequence (5′→3′)	Amplicon size (bp)	PCR conditions	Reference
*bla*CTX-M-group 1	F: GTTACAATGTGTGAGAAGCAG<br>R: CCGTTTCCGCTATTACAAAC	1017	94 °C 7 min<br>94 °C 50 s<br>50 °C 40 s (35 cycles)<br>68 °C 1 min<br>68 °C 5 min	[1]
*tet*A	F: GTAATTCTGAGCACTGTCGC<br>R: CTGCCTGGACAACATTGCTT	937	95 °C 5 min<br>95 °C 30 s<br>62 °C 30 s (23 cycles)<br>72 °C 45 s<br>72 °C 7 min	[2]
*tet*B	F: CTCAGTATTCCAAGCCTTTG<br>R: CTAAGCACTTGTCTCCTGTT	416	95 °C 5 min<br>95 °C 30 s<br>57 °C 30 s (25 cycles)<br>72 °C 20 s<br>72 °C 7 min	[2]
*sul*1	F: TGGTGACGGTGTTCGGCATTC<br>R: GCGAGGGTTTCCGAGAAGGTG	789	94 °C 5 min<br>94 °C 30 s<br>63 °C 30s (30 cycles)<br>72 °C 1 min<br>72 °C 8 min	[2]
*sul*2	F: CGGCATCGTCAACATAACC<br>R: GTGTGCGGATGAAGTCAG	722	94 °C 5 min<br>94 °C 30 s<br>50 °C 30 s (30 cycles)<br>72 °C 1.5 min	[2]

			72 °C 8 min	
*sul*3	F: CATTCTAGAAAACAGTCGTAGTTCG<br>R: CATCTGCAGCTAACCTAGGGCTTTGGA	792	94 °C 5 min<br>94 °C 1 min<br>51 °C 1 min (30 cycles)<br>72 °C 1 min<br>72 °C 5 min	[2]
*qnr*S	F: GCAAGTTCATTGAACAGGGT<br>R: TCTAAACCGTCGAGTTCGGCG	550	95 °C 5 min<br>95 °C 1 min<br>55 °C 1 min (35 cycles)<br>72 °C 1 min<br>72 °C 7 min	[3]
*qnr*A	F: AGAGGATTTCTCACGCCAGG<br>R: TGCCAGGCACAGATCTTGAC	580	95 °C 5 min<br>95 °C 1 min<br>55 °C 1 min (35 cycles)<br>72 °C 1 min<br>72 °C 7 min	[3]
*qnr*B	F: GGMATHGAAATTCGCCACTG<br>R: TTTGCYGYYCGCCAGTCGAA	264	95 °C 5 min<br>95 °C 1 min<br>55 °C 1 min (35 cycles)<br>72 °C 1 min<br>72 °C 7 min	[3]
*int*I1	F: GGGTCAAGGATCTGGATTTCG<br>R: ACATGGGTGTAAATCATCGTC	483	94 °C 5 min<br>94 °C 30 s<br>62 °C 30 s (30 cycles)<br>72 °C 1 min<br>72 °C 8 min	[2]

1. Jouini, A.; Vinue, L.; Slama, K. Ben; Saenz, Y.; Klibi, N.; Hammami, S.; Boudabous, A.; Torres, C. Characterization of CTX-M and SHV extended-spectrum -lactamases and associated resistance genes in *Escherichia coli* strains of food samples in Tunisia. *J. Antimicrob. Chemother.* **2007**, *60*, 1137–1141.

2. Sáenz, Y.; Briñas, L.; Domínguez, E.; Ruiz, J.; Zarazaga, M.; Vila, J.; Torres, C. Mechanisms of resistance in multiple-antibiotic-resistant *Escherichia coli* strains of human, animal, and food origins. *Antimicrob. Agents Chemother.* **2004**, *48*, 3996–4001.

3. Cattoir, V.; Poirel, L.; Rotimi, V.; Soussy, C.-J.; Nordmann, P. Multiplex PCR for detection of plasmid-mediated quinolone resistance qnr genes in ESBL-producing enterobacterial isolates. *J. Antimicrob. Chemother.* **2007**, *60*, 394–397.
